# Temporal proximity to counseling is associated with longitudinal bedaquiline and antiretroviral therapy adherence in multidrug-resistant tuberculosis and HIV treatment

**DOI:** 10.1371/journal.pone.0355696

**Published:** 2026-10-05

**Authors:** Xuan Lu, Jennifer Zelnick, Meng Zhao, Matthew Cummings, Allison K. Wolf, Kevin Guzman, Hlengiwe Nyilana, Rubeshan Perumal, Kathleen Rivet Amico, Karl Reis, Mbali Zulu, Amrita Daftary, Boitumelo Seepamore, Kogieleum Naidoo, Max O’Donnell

**Affiliations:** 1 Division of Pulmonary, Allergy, and Critical Care Medicine, Columbia University Irving Medical Center, New York, New York, United States of America; 2 Graduate School of Social Work, Touro University, New York, New York, United States of America; 3 School of Applied Human Sciences, University of KwaZulu-Natal, Durban, South Africa; 4 Department of Industrial and Systems Engineering, Lehigh University, Bethlehem, Pennsylvania, United States of America; 5 CAPRISA MRC- HIV-TB Pathogenesis and Treatment Research Unit, Durban, South Africa; 6 Department of Pulmonology and Critical Care, School of Clinical Medicine, College of Health Sciences, University of KwaZulu-Natal, Durban, South Africa; 7 University of Michigan School of Public Health, Ann Arbor, Michigan, United States of America; 8 Department of Medicine, University of Washington, Seattle, Washington, United States of America; 9 School of Global Health and Dahdaleh Institute of Global Health Research, York University, Toronto, Canada; 10 Department of Epidemiology, Columbia University Irving Medical Center, New York, New York, United States of America; Philadelphia University, JORDAN

## Abstract

**Background:**

Medication adherence is a persistent challenge in the treatment of multidrug-resistant tuberculosis (MDR-TB) and HIV. Routine counseling notes are an underutilized source of information on patient adherence context.

**Methods:**

We applied natural language processing (NLP) to routine counseling notes from a randomized controlled trial of MDR-TB/HIV treatment in South Africa. Topics were derived using topic modeling; notes were semantically matched to topics and clustered using K-means. We fitted linear mixed-effects (LME) regression models with participant-level random intercepts and five-fold cross-validation to model adherence to bedaquiline (BDQ) and antiretroviral therapy (ART), measured using cellular-enabled electronic dose monitor (EDM). Cosine similarity scores, cluster assignments, and temporal proximity to counseling sessions were included as covariates. Model fit was assessed using mean absolute error (MAE) and root mean square error (RMSE).

**Results:**

We analyzed 327 counseling notes from 76 participants. Six thematic clusters were derived: substance use, logistical barriers, limited social support, side effect management, financial stability, and financial insecurity/low support. LME showed modest fit. For BDQ adherence, MAE was 0.158 (95% CI 0.113–0.204) and RMSE 0.208 (95% CI 0.133–0.283). For ART, MAE was 0.180 (95% CI 0.170–0.191) and RMSE 0.224 (95% CI 0.202–0.246). Cluster membership was not associated with adherence. In contrast, temporal proximity to counseling was more consistently associated with adherence. Adherence was highest immediately following counseling sessions. Both BDQ and ART adherence declined during the intermediate period (β=−0.048, p = 0.001; β=−0.031, p = 0.022, respectively), and ART adherence declined further in the period preceding the next session (β=−0.032, p = 0.020).

**Conclusion:**

NLP methods structured routine counseling notes into interpretable, adherence-related themes. Temporal proximity to counseling sessions was consistently associated with longitudinal BDQ and ART adherence. These findings offer preliminary insights into adherence dynamics and generate hypotheses about the role of counseling session timing in adherence support.

## Introduction

Tuberculosis (TB) is a major global public health threat, with an estimated 10.8 million people diagnosed worldwide in 2023, and increasing global TB incidence since 2020 [1]. South Africa is among the ten countries classified by the World Health Organization as high-burden for TB, multidrug- or rifampicin-resistant TB (MDR/RR-TB), and TB/HIV co-infection [1]. South Africa was one of the first countries to implement a novel six-month bedaquiline-based MDR-TB regimen [2]. For MDR/RR-TB, six-month bedaquiline, pretomanid, and linezolid (BPaL)-based regimens have demonstrated high success rate of 90% in clinical trials [3]. However, in operational settings, these results have not been consistently replicated, which has been attributed to suboptimal medication adherence [4].

Medication adherence may be impacted by both structural and psychosocial barriers, including limited healthcare access, food insecurity, drug side effects, pill burden, substance use, and stigma [2,5–9]. For individuals living with HIV, concomitant antiretroviral therapy (ART) further challenges medication adherence [1,4,10,11]. Population specific intervention strategies to support ART adherence among people being treated for HIV, including peer support, SMS/phone call reminders, and adherence counseling have been developed alongside effective medication [12]. Intervention strategies to support TB medication adherence, including nutritional support and counseling, have also shown promising results [5,13,14].

Cellular-enabled electronic dose monitoring (EDM) devices, which can capture granular pill-taking behaviors, have been shown to accurately reflect treatment response and outcome in MDR-TB [15]. These devices typically share core functions, including automatic dose recording and patient reminder systems, and have demonstrated effectiveness in measuring adherence across multiple clinical studies [16–19]. However, results of studies to assess real-world use of EDM to support adherence are mixed, suggesting that technological solutions alone may be insufficient to address adherence challenges [20].

Qualitative data from routine care, such as medical encounters and counseling session notes, may offer critical insights into psychosocial factors shaping patient treatment experiences that can guide the use of appropriate targeted interventions to address specific adherence barriers [14,21]. Yet these data are often underutilized due to their unstructured format, a challenge often observed in low- and middle-income country settings, where the qualitative research capacity may be limited [21,22]. Advances in artificial intelligence, particularly natural language processing (NLP), now enable scalable, efficient analysis of these narratives [23,24]. NLP approaches, ranging from rule-based and machine learning methods to modern transformer architectures, can identify key themes, relationships, and contextual meanings within clinical text [25–27]. Such tools hold particular promise in resource-limited settings like South Africa, where data scarcity, infrastructure constraints, and high analytic costs hinder traditional qualitative research [28–30].

We applied natural language processing to routine adherence counseling notes from people with MDR-TB/HIV co-infection in South Africa to identify adherence-related themes and examine their longitudinal association with EDM-measured adherence to bedaquiline and antiretroviral therapy. We also included temporal proximity to counseling sessions as a covariate, to assess whether session timing explained adherence dynamics beyond thematic note content. Together, these analyses aimed to explore a scalable approach for structuring routinely collected clinical text to better understand adherence challenges in resource-limited settings.

## Methods

### Study design

Eligible participants were adults with confirmed MDR-TB and HIV, starting a bedaquiline (BDQ) -based regimen while on antiretroviral therapy (ART), and were prospectively enrolled to the PRospective study of Adherence in M/XDR-TB Implementation Science study (the PRAXIS study; ClinicalTrials.gov IDs: NCT03162107, NCT04032730) at a TB referral hospital and affiliated clinics in KwaZulu-Natal, South Africa. Participants were followed monthly for six months, and subsequently until treatment completion.

This study followed a proposed analysis framework (Fig 1).

**Fig 1:**
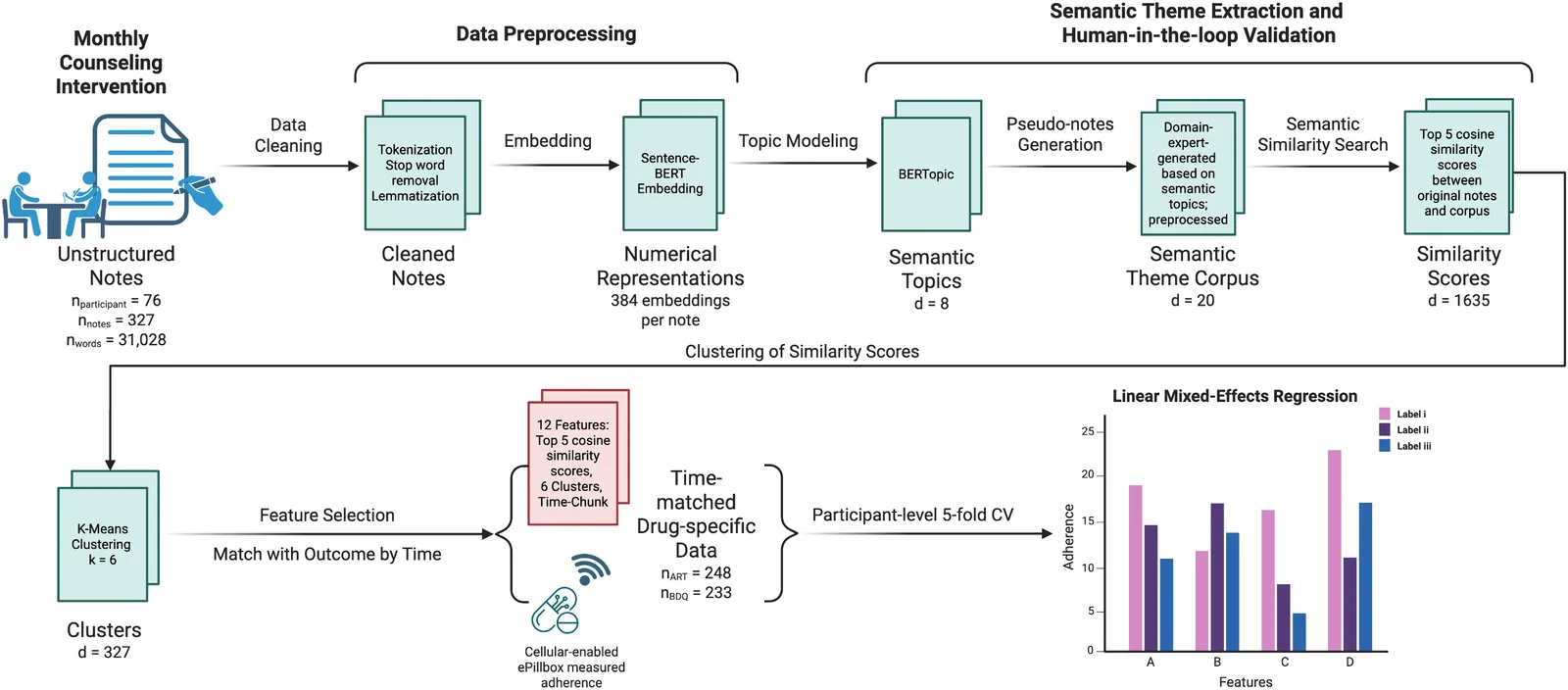
Analysis framework. Structured approach to extracting semantic features from unstructured counseling notes and using them to model medication adherence. *n* is sample size; *d* is number of variables.

### Data collection

At each follow-up visit, participants were scheduled to receive a monthly counseling intervention (MCI). The MCI followed a motivational interviewing framework and was guided by the situated Information, Motivation and Behavioral Skills (sIMB) model, with each session structured around a patient-developed personal adherence plan [31,32]. Each MCI session averaged 40 minutes and was conducted by two bilingual counselors in *isiZulu*. After the session, the counselors summarized their accounts of patient experiences and adherence barriers in *English*, following a semi-structured documentation template. These summary notes reflect interpreted rather than direct patient accounts, with potential for translation loss, documentation bias, and unmeasured counselor-level variation.

Adherence to both BDQ and ART was monitored using EDM devices (Wisepill RT2000). Each participant received separate devices for BDQ and ART at treatment initiation. Device activity was tracked in real time with missed openings serving as a surrogate for medication nonadherence.

### Data processing and exploratory analysis

The MCI notes were processed using a standardized preprocessing pipeline to optimize data for NLP analyses. Preprocessing involved tokenization, stop-word removal, and lemmatization. Preprocessed textual data were transformed into numerical representations using Sentence-BERT (SBERT), a transformer-based approach known for capturing semantic context [33]. SBERT embeddings provided dense, high-dimensional semantic representations of counseling notes and served as the foundational dataset for the subsequent topic modeling and semantic clustering analyses.

We temporally aligned adherence data to the timing of each participant’s MCI session and divided the interval between consecutive sessions into three categorical temporal periods (“time-chunks”): “Post-Session,” the first seven days following an MCI session; “Intermediate,” the period between the Post-Session and Pre-Session windows; and “Pre-Session,” the seven days preceding the subsequent MCI session. As initial exploratory analysis, we assessed the patterns of BDQ/ART adherence and the time-chunks using appropriate non-parametric tests.

### Topic modeling, semantic search and clustering

We used BERTopic coupled with clinical expert review to identify latent topics within the notes [34]. BERTopic is an unsupervised topic-modeling framework combining dimensionality reduction (Uniform Manifold Approximation and Projection, UMAP), clustering techniques (Hierarchical Density-Based Spatial Clustering of Applications with Noise, HDBSCAN), and keyword extraction (class-based term frequency-inverse document frequency, c-TF-IDF). After BERTopic returned latent keyword groups, we used these groups to construct a semantic reference corpus (“corpus”). Each sentence of the corpus is a pseudo-sample of counseling notes and represents a distinct topic (e.g., substance use, side effect, social support) from the keyword groups. We performed semantic search by computing the cosine similarity score between each of the notes and each of the sentences in the corpus. We then applied K-Means clustering to group cosine similarity scores into semantic clusters (“clusters”). We iteratively validated the latent topics and clusters with human-in-the-loop approach and used C_v_ (human interpretability score) to evaluate clustering results [35,36].

### Longitudinal association analyses

Only notes with available adherence follow-up data were included in the longitudinal association analyses. We obtained the top five cosine similarity scores of each note and their cluster assignments, mapped to time-chunks so the model may capture temporal dependencies. Mixed-effects modeling of adherence included fixed effects for semantic similarity scores (top 5), time-chunk (categorical), and cluster membership (categorical), with random intercepts for each participant to account for individual heterogeneity in baseline adherence. Model performance was evaluated using five-fold cross-validation and was assessed using mean absolute error (MAE), root mean squared error (RMSE), and R^2^. Splits were performed at participant-level to prevent data leakage and ensure independence between datasets. The final model was fitted on the complete dataset to obtain stable coefficient estimates for interpretation.

### Ethical considerations

The PRAXIS study, including data collection and all subsequent analyses, was approved by the Columbia University Irving Medical Center institutional review board (IRB-AAAQ5753) and University of KwaZulu-Natal Biomedical Research Ethics Committees (BE242/16). Written informed consent was obtained from all participants. Throughout the study, ethical standards were maintained to ensure patient confidentiality, secure data management, and compliance with all applicable regulations governing clinical research.

## Results

### Data processing and exploratory analysis

A total of 327 notes (31,028 words) from 76 participants, averaging 4 MCI sessions per participant, collected between April 2017 to September 2020, were included in the present analysis (Table 1). At data processing, SBERT embedding resulted in 384 dimensions of numerical representations. After temporal alignment, the dataset for longitudinal association analyses consisted of 233 counseling notes (22,729 words) from 71 participants for BDQ adherence, and 248 counseling notes (24,122 words) from 73 participants for ART adherence.

**Table 1:** Demographics.

Demographic Characteristic	N = 76^*1*^
Age	37 (30, 46)
Gender	
Male	44 (58%)
Female	32 (42%)
Education	
Primary or less	13 (17.1%)
Secondary	60 (79%)
College/University	3 (3.9%)
Marital Status	
Single	38 (50%)
Partner/Girlfriend/Boyfriend	29 (38%)
Married	6 (7.9%)
Divorced/Separated/Widowed	3 (3.9%)
Use of Alcohol/Smoking/Substance	52 (68%)
Have Children	60 (79%)
Number of Children	2.00 (1.00, 3.00)
Live in Informal Settlement	9 (12%)
Imprisonment History	8 (11%)
Employed	22 (29%)
Income (Rand)	2,815 (1,550, 4,450)
Number of household members supported by this income	5.00 (3.50, 7.00)
Social Grants [Missing: 2]	21 (28%)
Travel Time to Clinic (minutes) [Missing: 51]	45 (20, 60)
Travel Cost to Clinic (Rand) [Missing: 52]	40 (20, 49)

^*1*^Median (Q1, Q3); n (%).

Initial exploratory analysis on adherence by time-chunk with Kruskal-Wallis rank sum test and Dunn’s test found that Post-Session and Pre-Session have higher average adherence than Intermediate, for both BDQ and ART, and both were statistically significant (p < 0.01, p < 0.0001, respectively; Bonferroni-adjusted).

### Topic modeling, semantic search and clustering

BERTopic identified twenty latent keyword groups across the notes, with the top six groups demonstrating the most clarity and representativeness (S1 Table). We used the keyword groups to generate a twenty-sentence semantic reference corpus (“corpus”) (S1 Notes) for performing semantic search, in which we calculated the cosine similarity score between each note and each of the sentences in the corpus. We applied k-means clustering to the cosine similarity scores and examined solutions for k = 2–15. While statistical metrics favored lower k (k = 2–3), we selected k = 6, in consultation with content-domain experts (M.O., J.Z.), to prioritize clinical interpretability (k = 6; C_v_ = 0.689; Silhouette = 0.120) (Fig 2; S1A,B Fig). As an exploratory analysis, six clusters allowed sufficient granularity to extract clinically interpretable insights with modest semantic coherence.

**Fig 2:**
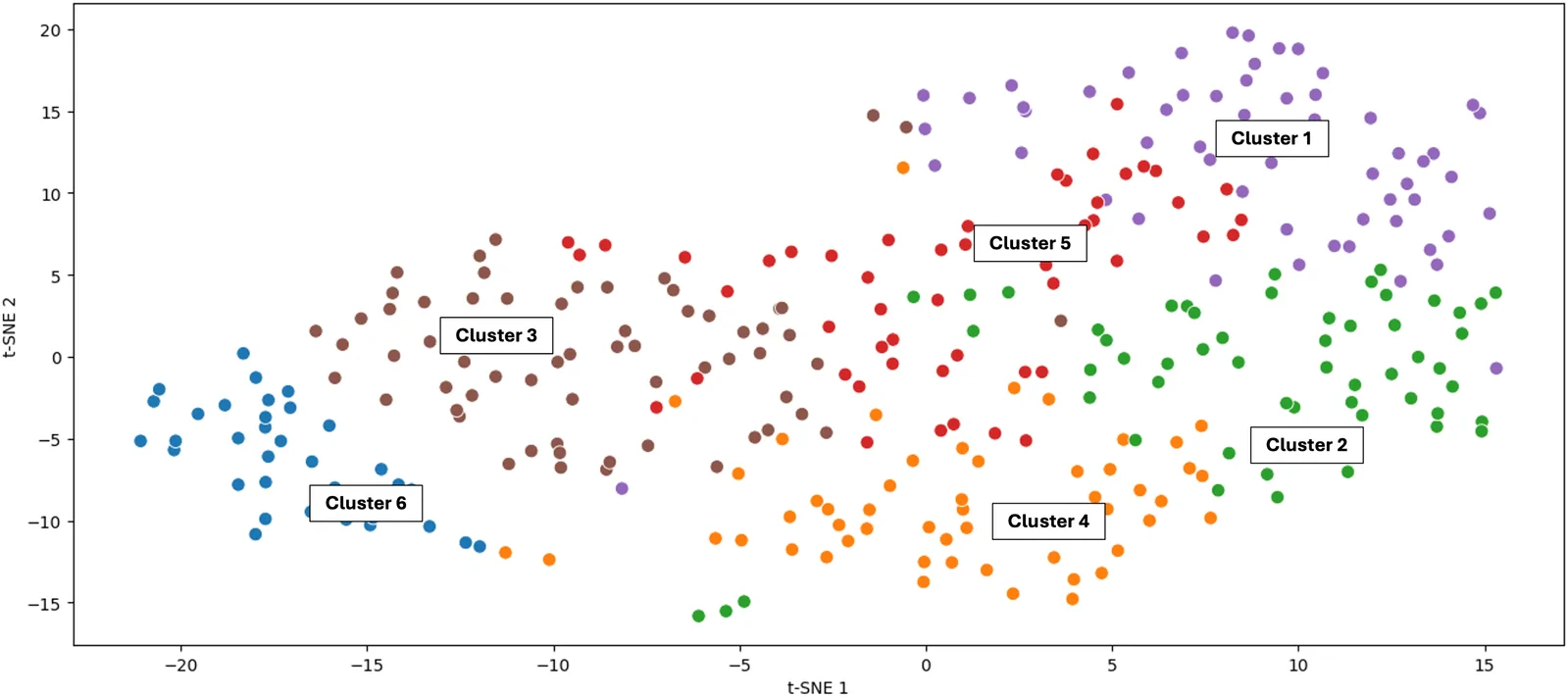
t-SNE plot of semantic clusters derived from K-means clustering (k = 6). K-means clustering of cosine similarity score of each note to each sentence in semantic reference corpus, which was generated from BERTopic-extracted latent topics.

For each cluster, clinical and social science experts (M.O. and J.Z.) reviewed the cluster center and the top three most frequently matched sentence from the corpus (S2 Table), and summarized semantic themes of each cluster for interpretability (Table 2). Notably, three corpus statements that frequently matched these clusters relate to dimensions of support from our motivational interviewing approach [31], informed by the sIMB model [32]: (1) “Patient received positive reinforcement for improved adherence this month.”, reflecting motivation through positive reinforcement; (2) “Counselor emphasized setting reminders and establishing a routine for medication.”, representing adherence strategies and skill building; and (3) “The session ended with affirming patient’s efforts and planning next steps.”, illustrating adherence planning and summarizing. These statements exemplify how key elements of the intervention aligned with our theoretical framework and were reflected in participant-counselor interactions.

**Table 2:** Cluster semantic themes from k-means clustering of semantic similarity scores, with human-in-the-loop validation.

Cluster	Semantic Theme
1	Adherence skills for alcohol/substance use challenges
2	Strategies to overcome logistical barriers to clinic attendance
3	Supporting patients in rural areas with limited social support
4	Skills to manage side effects to improve care engagement
5	Financial stability and medication adherence improvement
6	Strategies to overcome financial insecurity and low social support

### Longitudinal association analyses

Linear mixed-effects regression had the best performance across methods and was selected as our final modeling method (S3 Table). Using the top five cosine similarity scores, time-chunks, and cluster assignment as features, we fitted a linear mixed-effects regression model with participant random intercept to examine their longitudinal association with EDM-measured BDQ and ART adherence. Model performance across five-fold cross validation showed modest overall performance for both treatment types (S4 Table). For BDQ, the MAE was 0.158 (95% CI=[0.113, 0.204]), representing an average absolute error of 15.8 percentage points. For ART, the MAE was 0.180 (95% CI=[0.170, 0.191]), representing an average absolute error of 18.0 percentage points. The corresponding RMSEs were 0.208 (95% CI=[0.133, 0.283]) for BDQ and 0.224 (95% CI=[0.202,0.246]) for ART. These results suggest that the model partially tracked individual adherence patterns for both BDQ and ART, with slightly higher variability in residual variability in ART estimates compared to BDQ.

Cluster membership was not independently associated with adherence for either BDQ or ART. For BDQ, compared to cluster 1(substance use challenges), Clusters 2 (strategies to overcome logistical barriers), 5 (financial stability/adherence improvement), and 6 (financial insecurity/low social support) showed positive associations with adherence, while Clusters 3 (patient lives in rural areas/limited social support) and 4 (managing side effects) showed negative associations; however, none reached statistical significance (all p > 0.05), and the coefficient estimates are close to null. For ART, all clusters showed positive associations relative to Cluster 1 (substance use challenges) with larger coefficient estimates, though only Cluster 2 (strategies to overcome logistical barriers) achieved marginal statistical significance (p < 0.1).

Among the five semantic similarity scores, only the fourth similarity score was associated with ART adherence (β = 1.215, 95% CI=[0.318, 2.113], p = 0.008). However, this feature lacks direct clinical interpretability as it represents similarity to a composite theme rather than a specific counseling topic.

Time-chunk emerged to consistently associated with both medications. Relative to the immediate post-session period (first 7 days after counseling), adherence was significantly lower during the intermediate period for both BDQ (β = −0.048, 95% CI=[−0.075, −0.020], p = 0.001) and ART (β = −0.031, 95% CI=[−0.058, −0.005], p = 0.022). Additionally, ART adherence was significantly lower during the pre-session period (7 days before next counseling visit) compared to the post-session period (β = −0.032, 95% CI=[−0.059, −0.005], p = 0.020) (Fig 3; S5 Table).

**Fig 3:**
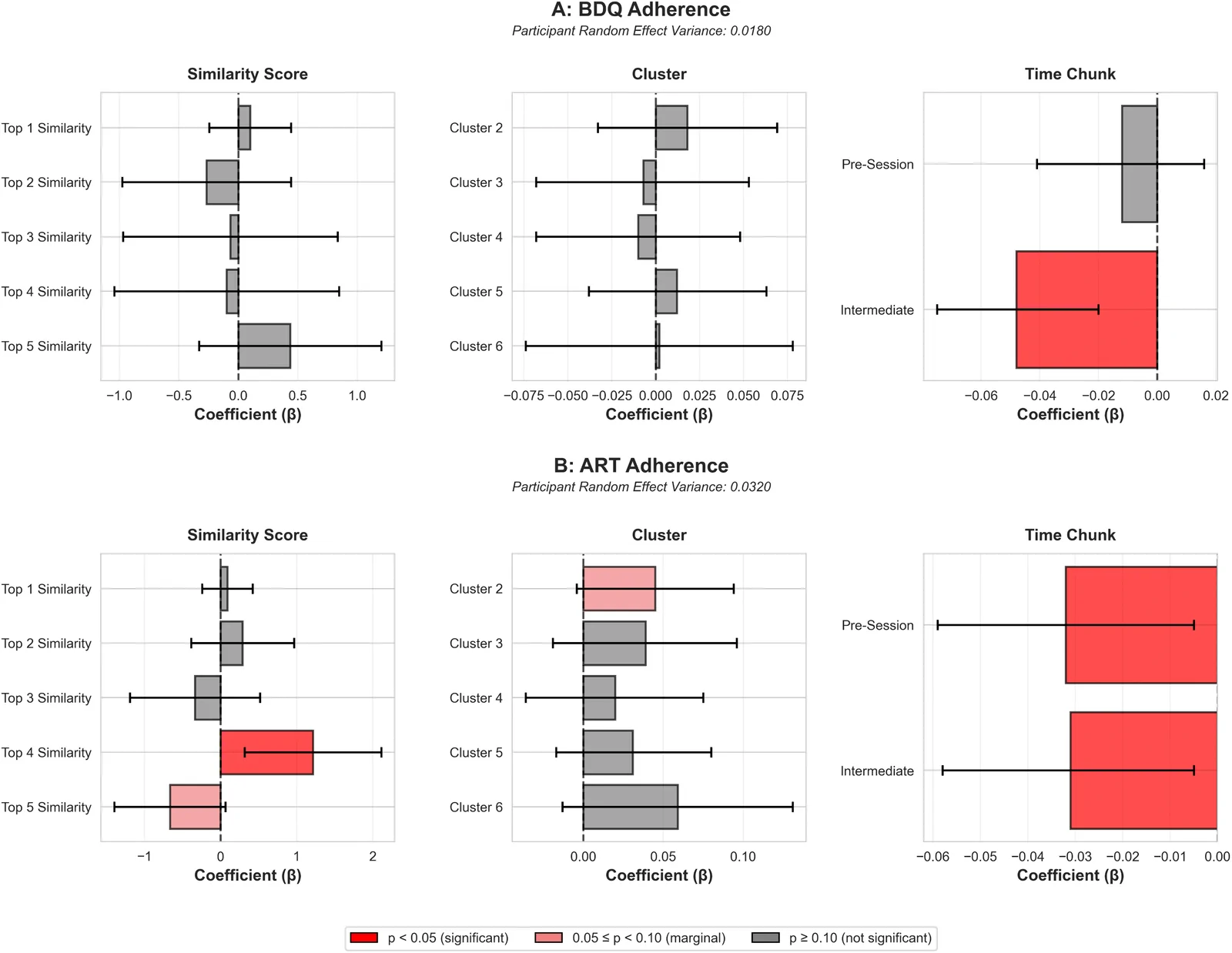
Fixed effects from linear mixed-effects models estimating associations with adherence to (A) BDQ and (B) ART. Bars represent coefficient estimates with 95% confidence intervals. Covariates include semantic similarity scores (Top 1-5 Similarity), k-means cluster assignment (Cluster 1-6; ref: Cluster 1), and time-relative periods within each MCI inter-visit interval (Post-Session, Intermediate, Pre-Session; ref: Post-Session). Color indicates statistical significance: red (p < 0.05), light coral (0.05 ≤ p < 0.10), gray (p ≥ 0.10). Models included participant-level random intercepts to account for individual heterogeneity (variance shown for each model). Positive coefficients indicate higher estimated adherence; negative coefficients indicate lower estimated adherence relative to the reference category.

To examine temporal variation in cluster effects, we fitted a secondary model with cluster x time-chunk interaction term. The interaction term between cluster 2 (strategies to overcome logistical barriers) and Pre-Session was associated with lower adherence to BDQ (β = −0.107; 95% CI=[−0.198, −0.017]; p = 0.021) (S6 Table).

## Discussion

Our analysis suggests that natural language processing of routine counseling notes, followed by longitudinal mixed-effects modeling, can identify adherence-related patterns in patients treated for MDR-TB and HIV. Although interpretable social and behavioral domains were derived in BERTopic and k-means clustering, cluster membership was not independently associated with EDM-measured longitudinal adherence. Instead, temporal proximity to counseling sessions emerged as robustly associated with adherence for BDQ and ART, with adherence declining during intermediate and pre-session intervals. These findings suggest that adherence patterns may not be driven by static thematic content of adherence counseling, but rather by time-dependent effects surrounding patient-counselor engagement. Collectively, our results suggest the utility of applying computational methods to routine clinical documentation and point to the importance of temporality as a target for hypothesis generation in future adherence research in high-burden treatment settings.

Our findings extend prior work applying NLP to medication adherence in chronic diseases, where structured and unstructured clinical documentation has been used to identify adherence risk and behavioral phenotypes [37,38], by demonstrating that similar methods can organize counseling documentation into descriptive, adherence-relevant themes. Prior NLP-based adherence studies have commonly relied on patient self-reporting, electronic health record metadata or claims data in high-resource settings [23,38,39]. In contrast, few NLP studies have used cellular-enabled EDM as an objective surrogate for adherence or examined unstructured counseling documentation, and none to our knowledge have done so in the context of MDR-TB/HIV co-treatment in high-burden settings. Adherence counseling interventions in MDR-TB and HIV fall under three main categories: educational, psychosocial, and motivational interviewing approaches [40]. These interventions have demonstrated variable impact with mixed evidence regarding which components of counseling are most effective [12,40,41]. Our results suggest, as a hypothesis for future investigation, that the timing of counseling interactions may be more consequential than specific thematic domains captured in session notes. This observation aligns with behavioral adherence models emphasizing reinforcement, accountability, direct support, and sustained engagement over discrete informational content [18].

Our study has several strengths. We combined NLP methods with longitudinal mixed-effects modeling to analyze routine counseling notes and examine their longitudinal associations with adherence to BDQ and ART among patients with MDR-TB and HIV. Prospective data collection within a randomized controlled trial reduced retrospective documentation bias and improved data quality compared to retrospective observational designs. The study was conducted within a public health infrastructure in a high-burden TB and HIV setting, enhancing the generalizability of findings to similar programmatic settings. Using cellular-EDM devices, we captured adherence behavior in real time and temporally aligned adherence with counseling encounters to examine longitudinal associations rather than cross-sectional relationships. We developed a structured analytic pipeline combining topic modeling, semantic similarity scoring, and clustering for thematic content extraction, followed by mixed-effects regression to assess longitudinal associations. Clinical expert review was incorporated throughout to ensure thematic coherence and clinical interpretability of derived clusters.

Our study has limitations. First, counseling sessions were conducted in *isiZulu* and documented in *English*, potentially introducing linguistic compression, interpretive bias, and documentation bias arising from counselor-level variation. The absence of counselor identifiers precluded assessment of counselor-level variation in documentation practices and its potential influence on thematic content, although semi-structured documentation format partially mitigated this concern. Second, our sample size, while substantial for an RCT in the study population, is modest compared to large-scale open-source corpora. This may limit statistical power, clustering performance, the stability of model estimates, and may suffer from overfitting. Third, the study context focused on TB/HIV care in South Africa and may reduce the generalizability of the findings to other populations or settings. External validation in an independent, ideally multi-site cohort therefore represents an important direction for future work. Fourth, the six-cluster solution was selected as an exploratory descriptive framework based on interpretability rather than cluster validity metrics alone. Clusters exhibited modest coherence based on silhouette scores, potentially reflecting limited sample size, intrinsic overlap in the underlying semantic structure, or both, and should not be interpreted as a strongly separated or uniquely data-driven latent structure. Instead, the clusters should be interpreted as thematic organization of collected counseling notes rather than robust latent phenotypes. Furthermore, silhouette scores may not accurately capture cluster coherence in NLP contexts, where semantic similarity and high-dimensional embeddings may compress inter-cluster distances, reducing the metric’s discriminative power. Fifth, since adherence measure is bounded between 0 and 1, model estimates may be subject to ceiling effects and regression toward the mean, particularly in cases of extreme adherence behavior. Lastly, our study represents an exploratory longitudinal association analysis rather than a clinically deployable prediction tool, and findings should be interpreted as preliminary evidence of feasibility and a basis for hypothesis generation rather than as grounds for practice change or intervention guidance.

## Conclusion

Analyzing routine adherence counseling notes using NLP and longitudinal mixed-effects modeling provided insight into adherence dynamics among patients with MDR-TB and HIV. Although thematic content derived through exploratory clustering was not independently associated with adherence, temporal proximity to counseling sessions was consistently associated with adherence to BDQ and ART. These findings provide preliminary evidence of the feasibility of applying NLP methods to routine counseling documentation and generate hypotheses about the role of counseling session timing in adherence dynamics. Future studies should evaluate these methods in larger and more diverse populations and examine the mechanisms by which counseling session proximity influences adherence behavior, and evaluate generalizability across varied clinical settings.

## Supporting information

S1 Notes Semantic theme corpus generated from BERTopic results. (DOCX)

S1 Fig Semantic clusters derived from k-means clustering (k = 6). (A) K-optimization and (B) Silhouette plot of k-means clustering of cosine similarity score of each note to each sentence in semantic corpus, which was generated from BERTopic-extracted latent topics.
(TIF)

S1 Table Top 6 topics from BERTopic, with human-in-the-loop validation. (DOCX)

S2 Table Cluster center and most frequently matched semantic themes from semantic similarity search. (DOCX)

S3 Table Modeling method performance comparison. (DOCX)

S4 Table Linear mixed-effects regression model cross-validation results. (DOCX)

S5 Table Linear mixed-effects model coefficients. (DOCX)

S6 Table Cluster and time-chunk interaction term coefficients in secondary model. (DOCX)
