# Supplementary material for: Temporal proximity to counseling is associated with longitudinal bedaquiline and antiretroviral therapy adherence in multidrug-resistant tuberculosis and HIV treatment
Source: PLoS One. 2026 Oct 5;21(10):e0355696. doi: 10.1371/journal.pone.0355696 (PMC13637884; doi:10.1371/journal.pone.0355696)
Supplement: Supplementary file 1 [file pone.0355696.s001.docx]

**S1 Notes. Semantic theme corpus generated from BERTopic results.**

1. Patient experienced side effects like nausea, fatigue, painful joints and feet, poor vision, and blindness.

2. Fear of community stigma discouraged patient from attending clinic appointments.

3. Patient expressed determination to complete TB treatment and stay healthy.

4. Challenges in remembering doses led to missed medication days this week.

5. Patient described food insecurity as a barrier to taking medication regularly.

6. Living in rural area and poor internet connection caused Wisepill device to not record intakes.

7. Patient disclosed alcohol use interfering with evening medication intake.

8. Counselor emphasized setting reminders and establishing a routine for medication.

9. Patient received positive reinforcement for improved adherence this month.

10. Missed appointments were due to transport issues and clinic wait times.

11. Patient shared feelings of isolation and lack of emotional support at home.

12. Mental health concerns, including depression and anxiety, were discussed.

13. Counselor helped identify treatment goals and steps toward long-term adherence.

14. Support group sessions were discussed as a source of encouragement.

15. Referral was made to social worker to address financial and housing needs.

16. Patient explained confusion about pill timing and dosage instructions.

17. Motivational interviewing was used to build confidence in treatment plan.

18. Side effects of ART and TB drugs were clarified and normalized.

19. Substance use and traditional medicine were discussed.

20. The session ended with affirming patient’s efforts and planning next steps.
