# Supplementary material for: Temporal proximity to counseling is associated with longitudinal bedaquiline and antiretroviral therapy adherence in multidrug-resistant tuberculosis and HIV treatment
Source: PLoS One. 2026 Oct 5;21(10):e0355696. doi: 10.1371/journal.pone.0355696 (PMC13637884; doi:10.1371/journal.pone.0355696)
Supplement: Supplementary file 2 [file pone.0355696.s003.docx]

**S1 Table. Top 6 topics from BERTopic, with human-in-the-loop validation.**

| **Topics** | **Keywords** | **Example from Counseling Notes** | **Count** |
| --- | --- | --- | --- |
| *Support experiences* | home, family, child, grant, partner, support, stay, currently, time, work | “The participant is happy to be at home. She said her family is very supportive; as a result, she was accompanied by her sister, who is her treatment supporter.” | 42 |
| *Medication side effects* | vomit, feel, effect, weak, physically, nausea, better, family | “The participant is physically weak. She said that for the past three weeks, she has had vomiting troubles.” | 29 |
| *Impacts of traditional medicines, alcohol and substance use* | adherence, substance, use, alcohol, effect, traditional medicine, study | “She wants to understand from the nurse regarding the effects of alcohol and traditional medicine, which she views as hindrances to adherence.” | 25 |
| *Barriers to clinic visit attendance* | clinic, covid, test, keep clinic appointment, prescribe, importance | “He missed his clinic visit last week because he went home due to the lockdown caused by COVID-19.” | 16 |
| *Barriers to care due to rural residence* | device, intake, rural area, problem, poor network, reside, home | “The patient said she did not face any challenges with the devices other than network issues, as she resides in a rural area.” | 15 |
| *Barriers to accessing social grants* | grant, sassa, application, letter, disability grant, clinician, adherence | “What is stressing her the most is that she can't apply for a grant because she can't get a letter from the department of labor to prove that she is not working.” | 14 |
| Note: Topics were ranked by the number of notes assigned to each topic. Keywords were ordered by their class-based TF-IDF (c-TF-IDF) scores and manually screened for interpretability. Low-utility words are put to the end and grayed out. Topics were ranked by “count”, the number of notes assigned. | | | |
