# Supplementary material for: Temporal proximity to counseling is associated with longitudinal bedaquiline and antiretroviral therapy adherence in multidrug-resistant tuberculosis and HIV treatment
Source: PLoS One. 2026 Oct 5;21(10):e0355696. doi: 10.1371/journal.pone.0355696 (PMC13637884; doi:10.1371/journal.pone.0355696)
Supplement: Supplementary file 3 [file pone.0355696.s004.docx]

**S2 Table. Cluster center and most frequently matched semantic themes from semantic similarity search.**

| **Cluster** | **Cluster Center (reverted to original texts)** | **First Frequently Matched** | **Second Frequently Matched** | **Third Frequently Matched** |
| --- | --- | --- | --- | --- |
| 1 | Participant mentioned that he was confused when called by CAPRISA^1^ staff about the devices because he ensures that he takes his medications every day. He was counseled about the importance of taking all his medications correctly and daily. He stated that he does take all his medications and was very upset about the calls he was receiving from CAPRISA, as it made it seem as if he was not adhering to his medication. I apologized and explained to him that our aim is to support him and to see if the device can help patients adhere to their medications. We believe that by working together, we can fight TB, and he understood. | Patient received positive reinforcement for improved adherence this month. | Counselor emphasized setting reminders and establishing a routine for medication. | Patient disclosed alcohol use interfering with evening medication intake. |
| 2 | An MCI was conducted with the patient, and she said she was doing well. However, since she started the treatment, she has been having side effects. She added that she still gets support at home. The patient was advised to continue taking her medication the way she has been taking them and continue using the devices. | Patient received positive reinforcement for improved adherence this month. | The session ended with affirming patient’s efforts and planning next steps. | Missed appointments were due to transport issues and clinic wait times. |
| 3 | The participant reported that he is feeling very well and has experienced no challenges with taking treatment from home. He said he always manages to take treatment on his own when he is at home in Kwa Maphumulo. Sometimes, he stays in Stanger, at his renting place. He usually goes to Shanksville to be closer to Stanger hospital, where he takes an ambulance from there to KDH^2^. He is still unemployed and receiving a SASSA^3^ grant. | The session ended with affirming patient’s efforts and planning next steps. | Patient received positive reinforcement for improved adherence this month. | Patient shared feelings of isolation and lack of emotional support at home. |
| 4 | The patient came for the ASG^4^, and an MCI was conducted. The patient said that she was doing fine but has a toothache, and the doctor said that she is going to be alright. She also added that even the side effects are much better; it is not like before. The only side effects she has are on her feet. The patient mentioned that at home, her family is very supportive, and they help her with her treatment. The patient was encouraged to continue taking her medication and using the devices, and she said that she will do so. | Patient experienced side effects like nausea, fatigue, painful joints and feet, poor vision, and blindness. | The session ended with affirming patient’s efforts and planning next steps. | Missed appointments were due to transport issues and clinic wait times. |
| 5 | This was the patient’s month 6 study visit. According to the dashboard, the patient has been consistent in taking his medication. The patient has also been vocal in the ASG sessions and often dominated the discussions. The patient seemed happy in this session and said his life has improved financially since getting the disability grant, which he will receive for 6 months. | Patient received positive reinforcement for improved adherence this month. | The session ended with affirming patient’s efforts and planning next steps. | Counselor emphasized setting reminders and establishing a routine for medication. |
| 6 | According to the dashboard, the participant is doing well on treatment, and he states that he is committed to adhering to treatment. He says that he had cramps in the feet and reported this to the doctor, who then gave him tablets. He also said his disability grant application was difficult, but eventually, it was successful. He will be getting his first payment in January and says this will assist him and his family financially. | Referral was made to social worker to address financial and housing needs. | Patient shared feelings of isolation and lack of emotional support at home. | The session ended with affirming patient’s efforts and planning next steps. |

**Note:** ^1^Centre for the Aids Programme of Research In South Africa; ^2^King Dinuzulu Hospital, Durban, South Africa; ^3^South African Social Security Agency; ^4^Adherence Support Group
