# Supplementary material for: Temporal proximity to counseling is associated with longitudinal bedaquiline and antiretroviral therapy adherence in multidrug-resistant tuberculosis and HIV treatment
Source: PLoS One. 2026 Oct 5;21(10):e0355696. doi: 10.1371/journal.pone.0355696 (PMC13637884; doi:10.1371/journal.pone.0355696)
Supplement: Supplementary file 4 [file pone.0355696.s005.docx]

**S3 Table. Modeling method performance comparison.**

| **Model** | **MAE mean (SD)** | **RMSE mean (SD)** | **R2 mean (SD)** |
| --- | --- | --- | --- |
| **A. BDQ** | | | |
| Baseline Median | 0.11 (0.048) | 0.228 (0.066) | NA (NA) |
| Baseline Mean | 0.16 (0.029) | 0.206 (0.054) | NA (NA) |
| Linear Mixed Effects | 0.155 (0.037) | 0.207 (0.06) | -0.088 (0.052) |
| Generalized Additive Model | 0.161 (0.03) | 0.213 (0.051) | -0.166 (0.154) |
| LSTM | 0.146 (0.038) | 0.203 (0.056) | -0.035 (0.053) |
| Ridge Regression | 0.159 (0.031) | 0.21 (0.053) | -0.114 (0.057) |
| Random Forest | 0.167 (0.027) | 0.226 (0.044) | -0.348 (0.268) |
| **B. ART** | | | |
| Baseline Median | 0.158 (0.024) | 0.233 (0.041) | NA (NA) |
| Baseline Mean | 0.17 (0.01) | 0.216 (0.022) | NA (NA) |
| Linear Mixed Effects | 0.18 (0.008) | 0.224 (0.018) | -0.169 (0.223) |
| Generalized Additive Model | 0.172 (0.01) | 0.225 (0.025) | -0.155 (0.079) |
| LSTM | 0.156 (0.025) | 0.228 (0.037) | -0.177 (0.112) |
| Ridge Regression | 0.174 (0.008) | 0.223 (0.022) | -0.145 (0.113) |
| Random Forest | 0.175 (0.009) | 0.228 (0.016) | -0.215 (0.255) |
