# Supplementary material for: Temporal proximity to counseling is associated with longitudinal bedaquiline and antiretroviral therapy adherence in multidrug-resistant tuberculosis and HIV treatment
Source: PLoS One. 2026 Oct 5;21(10):e0355696. doi: 10.1371/journal.pone.0355696 (PMC13637884; doi:10.1371/journal.pone.0355696)
Supplement: Supplementary file 5 [file pone.0355696.s006.docx]

**S4 Table. Linear mixed-effects regression model cross-validation results.**

| **A. BDQ** | | | | | | | |
| --- | --- | --- | --- | --- | --- | --- | --- |
| **Fold** | **MAE** | **RMSE** | **R^2^** | **n train participants** | **n test participants** | **n train samples** | **n test samples** |
| **1** | 0.142 | 0.185 | -0.017 | 57 | 14 | 539 | 135 |
| **2** | 0.149 | 0.210 | -0.073 | 56 | 15 | 539 | 135 |
| **3** | 0.131 | 0.175 | -0.074 | 57 | 14 | 539 | 135 |
| **4** | 0.133 | 0.158 | -0.141 | 57 | 14 | 539 | 135 |
| **5** | 0.221 | 0.309 | -0.137 | 57 | 14 | 540 | 134 |
| **B. ART** | | | | | | | |
| **Fold** | **MAE** | **RMSE** | **R^2^** | **n train participants** | **n test participants** | **n train samples** | **n test samples** |
| **1** | 0.180 | 0.208 | -0.541 | 58 | 15 | 583 | 147 |
| **2** | 0.169 | 0.217 | -0.034 | 58 | 15 | 583 | 147 |
| **3** | 0.177 | 0.215 | 0.017 | 58 | 15 | 584 | 146 |
| **4** | 0.183 | 0.227 | -0.199 | 59 | 14 | 585 | 145 |
| **5** | 0.192 | 0.253 | -0.088 | 59 | 14 | 585 | 145 |
