# Supplementary material for: Temporal proximity to counseling is associated with longitudinal bedaquiline and antiretroviral therapy adherence in multidrug-resistant tuberculosis and HIV treatment
Source: PLoS One. 2026 Oct 5;21(10):e0355696. doi: 10.1371/journal.pone.0355696 (PMC13637884; doi:10.1371/journal.pone.0355696)
Supplement: Supplementary file 6 [file pone.0355696.s007.docx]

**S5 Table. Linear mixed-effects model coefficients.**

|  |  |  | **BDQ** |  | |  |  | **ART** |  |
| --- | --- | --- | --- | --- | --- | --- | --- | --- | --- |
|  | **β** | **SE** | **95% CI** | **p** | | **β** | **SE** | **95% CI** | **p** |
| **Intercept** | 0.887 | 0.114 | 0.663, 1.111 | **<0.001** | | 0.538 | 0.108 | 0.327, 0.750 | **<0.001** |
| **Top Similarity Scores** | | | | |  |  |  |  |  |
| Top 1 | 0.099 | 0.175 | -0.244, 0.442 | 0.573 | | 0.091 | 0.169 | -0.240, 0.422 | 0.589 |
| Top 2 | -0.267 | 0.362 | -0.977, 0.443 | 0.461 | | 0.290 | 0.345 | -0.385, 0.966 | 0.399 |
| Top 3 | -0.066 | 0.460 | -0.968, 0.836 | 0.887 | | -0.336 | 0.436 | -1.191, 0.519 | 0.441 |
| Top 4 | -0.098 | 0.482 | -1.043, 0.847 | 0.839 | | 1.215 | 0.458 | 0.318, 2.113 | **0.008** |
| Top 5 | 0.436 | 0.391 | -0.330, 1.203 | 0.265 | | -0.664 | 0.373 | -1.395, 0.066 | 0.075 |
| **Time-Chunk (ref = Post-Session)** | | | | |  |  |  |  |  |
| Intermediate | -0.048 | 0.014 | -0.075, -0.020 | **0.001** | | -0.031 | 0.014 | -0.058, -0.005 | **0.022** |
| Pre-Session | -0.012 | 0.014 | -0.041, 0.016 | 0.399 | | -0.032 | 0.014 | -0.059, -0.005 | **0.020** |
| **Clusters (ref = cluster 1)** | | | | |  |  |  |  |  |
| Cluster 2 | 0.018 | 0.026 | -0.033, 0.069 | 0.485 | | 0.045 | 0.025 | -0.004, 0.094 | 0.070 |
| Cluster 3 | -0.007 | 0.031 | -0.068, 0.053 | 0.816 | | 0.039 | 0.029 | -0.019, 0.096 | 0.186 |
| Cluster 4 | -0.010 | 0.030 | -0.068, 0.048 | 0.734 | | 0.020 | 0.028 | -0.036, 0.075 | 0.488 |
| Cluster 5 | 0.012 | 0.026 | -0.038, 0.063 | 0.629 | | 0.031 | 0.025 | -0.017, 0.080 | 0.207 |
| Cluster 6 | 0.002 | 0.039 | -0.074, 0.078 | 0.955 | | 0.059 | 0.037 | -0.013, 0.131 | 0.107 |
