# Supplementary material for: Temporal proximity to counseling is associated with longitudinal bedaquiline and antiretroviral therapy adherence in multidrug-resistant tuberculosis and HIV treatment
Source: PLoS One. 2026 Oct 5;21(10):e0355696. doi: 10.1371/journal.pone.0355696 (PMC13637884; doi:10.1371/journal.pone.0355696)
Supplement: Supplementary file 7 [file pone.0355696.s008.docx]

**S6 Table. Cluster and time-chunk interaction term coefficients in secondary model.**

| **A. BDQ** |  |  |  |  |
| --- | --- | --- | --- | --- |
| **Parameter** | **Coefficient** | **CI_Lower** | **CI_Upper** | **p_value** |
| **Intermediate x Cluster 2** | -0.077 | -0.172 | 0.018 | 0.111 |
| **Pre-Session x Cluster 2** | -0.037 | -0.133 | 0.060 | 0.454 |
| **Intermediate x Cluster 3** | -0.034 | -0.122 | 0.055 | 0.458 |
| **Pre-Session x Cluster 3** | -0.107 | -0.198 | -0.017 | 0.021 |
| **Intermediate x Cluster 4** | -0.022 | -0.116 | 0.073 | 0.654 |
| **Pre-Session x Cluster 4** | -0.039 | -0.135 | 0.057 | 0.427 |
| **Intermediate x Cluster 5** | -0.053 | -0.147 | 0.041 | 0.270 |
| **Pre-Session x Cluster 5** | -0.065 | -0.162 | 0.031 | 0.184 |
| **Intermediate x Cluster 6** | -0.055 | -0.156 | 0.046 | 0.285 |
| **Pre-Session x Cluster 6** | -0.065 | -0.169 | 0.039 | 0.222 |
| **B. ART** |  |  |  |  |
| **Parameter** | **Coefficient** | **CI_Lower** | **CI_Upper** | **p_value** |
| **Intermediate x Cluster 2** | -0.014 | -0.104 | 0.077 | 0.768 |
| **Pre-Session x Cluster 2** | -0.027 | -0.119 | 0.064 | 0.561 |
| **Intermediate x Cluster 3** | 0.020 | -0.066 | 0.105 | 0.654 |
| **Pre-Session x Cluster 3** | -0.017 | -0.104 | 0.070 | 0.699 |
| **Intermediate x Cluster 4** | -0.014 | -0.104 | 0.076 | 0.758 |
| **Pre-Session x Cluster 4** | -0.075 | -0.166 | 0.016 | 0.107 |
| **Intermediate x Cluster 5** | 0.022 | -0.069 | 0.112 | 0.643 |
| **Pre-Session x Cluster 5** | -0.065 | -0.157 | 0.028 | 0.172 |
| **Intermediate x Cluster 6** | 0.019 | -0.080 | 0.118 | 0.704 |
| **Pre-Session x Cluster 6** | -0.055 | -0.155 | 0.045 | 0.279 |

Note: Models were fitted with full dataset. No cross-validation was performed.
